# Puerarin Alleviates Depression-Like Behavior Induced by High-Fat Diet Combined With Chronic Unpredictable Mild Stress *via* Repairing TLR4-Induced Inflammatory Damages and Phospholipid Metabolism Disorders

**DOI:** 10.3389/fphar.2021.767333

**Published:** 2021-12-15

**Authors:** Li-Na Gao, Maocai Yan, Lirun Zhou, Jian’an Wang, Chunmei Sai, Yingjie Fu, Yang Liu, Lin Ding

**Affiliations:** ^1^ College of Pharmacy, Jining Medical University, Rizhao, China; ^2^ Shandong Collaborative Innovation Center for Diagnosis, Treatment and Behavioral Interventions of Mental Disorders, Institute of Mental Health, Jining Medical University, Jining, China; ^3^ Institute of Chinese Materia Medica, China Academy of Chinese Medical Sciences, Beijing, China

**Keywords:** puerarin, depression, Toll-like receptor 4, inflammation, cytosolic phospholipases A2, cyclooxygenase 2

## Abstract

Puerarin has been reported as a potential agent for neuro-inflammatory disorders. However, there have been no reports of using puerarin for the treatment of depression based on Toll-like receptor 4 (TLR4)–mediated inflammatory injury. In this study, we evaluated the protective effects of puerarin on depression-like rats induced by a high-fat diet (HFD) combined with chronic unpredictable mild stress (CUMS). The mechanism was screened by lipidomics and molecular docking and confirmed by *in vivo* tests. Puerarin treatment significantly improved 1% sucrose preference and ameliorated depression-like behavior in the open-field test. The antidepressive effects of puerarin were associated with decreased pro-inflammatory cytokine production, including interleukin-6 (IL-6) and tumor necrosis factor-α (TNF-α), and increased anti-inflammatory cytokine levels (IL-10) in rat hippocampal tissues and plasma. Hematoxylin–eosin (H&E), immunofluorescence staining, and Western blotting results displayed that puerarin alleviated inflammatory injury by suppressing TLR4 expression and by repairing the intestine mucus barrier *via* enhancing the expression of claudin-1 and occludin. Non-targeted lipidomics analysis showed that the most significantly different metabolites modified by puerarin were phospholipids. Puerarin treatment–altered biomarkers were identified as PC (15:1/20:1), PE (15:1/16:1), and PI (18:2/20:1) in comparison with the HFD/CUMS group. Molecular docking modeling revealed that puerarin could bind with cytosolic phospholipase A2 (cPLA2) and cyclooxygenase-2 (COX-2), which play central roles in TLR4-mediated phospholipid metabolism. *In vivo*, puerarin treatment decreased the enzyme activities of cPLA2 and COX-2, resulting in lower production of prostaglandin E_2_ (PGE_2_) in hippocampal and intestinal tissues. In conclusion, puerarin treatment reverses HFD/CUMS-induced depression-like behavior by inhibiting TLR4-mediated intestine mucus barrier dysfunction and neuro-inflammatory damages *via* the TLR4/cPLA2/COX-2 pathway.

## Introduction

Depression is characterized by long-term depressed mood and loss of interest and pleasure. The phenotype and course of a depressive episode is singular, recurring, or chronic (Gururajan et al., 2019). Thus, depression is highly heterogeneous. Currently, the proportion of the global population suffering from major depression has increased to more than 300 million people (World Health, 2017). In addition to the extraordinary economic burden, the tragedy of suicide contributes to the high mortality of this disorder. Moreover, a major depression-induced reduction in lifespan is due to the significant increase in vulnerability to major medical disorders, such as stroke, diabetes, cancer, and cardiovascular disease (Beurel et al., 2020). Several hypotheses have been developed for the pathologic process of depressive disorder, including the monoamine hypothesis (Hirschfeld, 2000), glutamate hypothesis (Sanacora et al., 2012), and hypothalamic–pituitary–adrenocortical (HPA) axis hypothesis (Holsboer, 2000). A reflection of the 20-year studies indicates that increased inflammation and hyperactivity of the HPA axis are two of the most consistent biological findings in major depression (Pariante, 2017). However, the underlying pathological mechanism is still elusive.

Inflammation plays a vital role in the pathophysiology of depression. In comparison with healthy controls, patients with major depression have higher concentrations of inflammatory cytokines, including tumor necrosis factor (TNF)-α, interleukin (IL)-1β, IL-6, and C-reactive protein (CRP), while lower IL-10 concentration in blood (Euteneuer et al., 2017; Felger et al., 2020). Pro-inflammatory cytokines may be produced in the brain or may enter the brain from the periphery through 1) active transport; 2) “leaky” area across the blood–brain barrier (BBB); and 3) the afferent vagal pathway or activated monocytes, generating second messenger signals to activate glial cells to overproduce cytokines. Abnormally heightened cytokines change the brain structure and function by affecting neurotransmission, HPA axis, hippocampal neurogenesis, etc. (Kempuraj et al., 2017; Cervellati et al., 2020). Moreover, cytokines may dysregulate the levels of serotonin transporter proteins, tryptophan 2,3-dioxygenase (TDO), indoleamine 2,3-dioxygenase (IDO), and endogenous metabolites (Kindler et al., 2020). Toll-like receptors, the innate immune response pattern recognition receptors, have been described along with pro-inflammatory signaling pathways. TLR4 predominantly recognizes lipopolysaccharides (LPS) from Gram-negative bacteria. Changes of TLR4 signaling pathways were found in the peripheral circulation system or the central nervous system (CNS) of patients with major depression and depression-like animal models (Hung et al., 2016; Rahimifard et al., 2017; Fu et al., 2019). Most important is that TLR4 is an independent risk factor related to the severity of depression (Wu et al., 2015). In addition to neuroimmune and neuroendocrine systems, upregulated TLR4 signaling in blood was associated with leakage of damage-related molecular patterns through the gut (Hung et al., 2016). Thus, the link between TLR4 and symptoms of depression should be further investigated, and TLR4 may be a potential therapeutic target for the development of antidepressants.

In chronic stress–induced depressive mice, hippocampal damages are triggered by activating TLR4, glycogen synthase kinase-3 (GSK3), phosphatidylinositol-3/protein kinase B (PI3K/AKT), and downstream inflammatory signal transmission (Cheng et al., 2016). Mice suffering from chronic stress are more likely to show depression-like behavioral phenotypes, and TLR4 knockout can reduce chronic stress–induced cytokine levels in the mice hippocampus (Cheng et al., 2016). In addition, chronic stress–induced animals show heightened intestinal permeability by activating the TLR4-related signal transduction pathways (Gárate et al., 2013; Guo et al., 2019). TLR4 is a key molecule that regulates dietary nutrition, intestinal flora, and metabolic inflammation. A long-term high-fat diet (HFD) damages the intestinal permeability and increases the content of toxins, such as LPS, in intestinal tissues (Velloso et al., 2015). LPS activates immune cells by binding to the CD14–TLR4 complex, leading to the transcription of nuclear factor-κB (NF-κB) to generate large amounts of TNF-α, IL-6, and oxygenase-2 (COX-2) and induce the inflammation cascade (Yiu et al., 2017). Accordingly, both HFD and chronic stress induce the activation of TLR4 in the intestine and central nervous system, resulting in the over-production of cytokines. TLR4-mediated inflammation may be an important node in the molecular network of diseases induced by an HFD and chronic stress.

**FIGURE 1:**
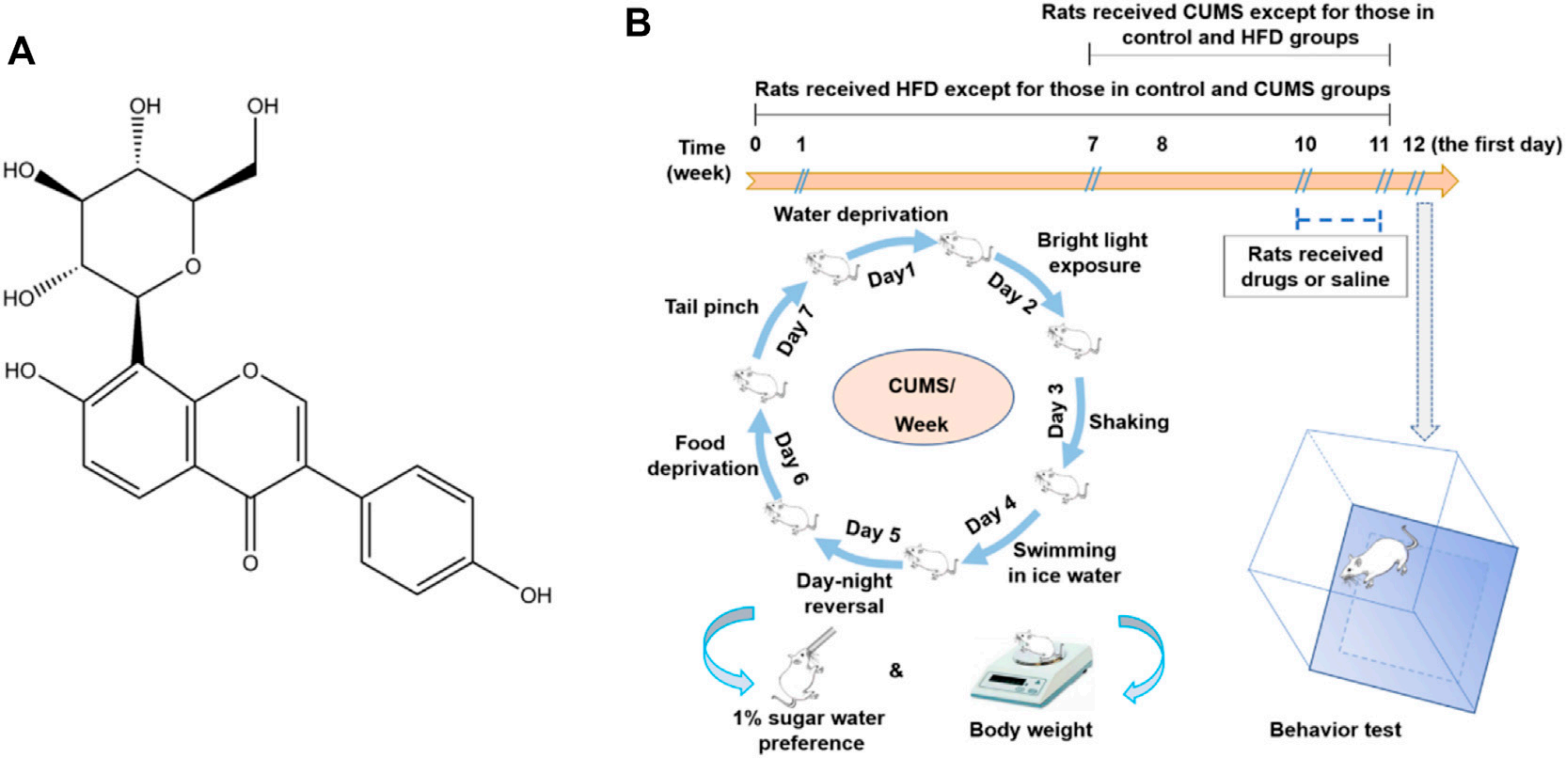
Structure of puerarin **(A)** and a schematic diagram of the HFD/CUMS procedure, drug administration, and behavioral evaluation **(B)**.

Chronic unpredictable mild stress (CUMS) simulates the social stress, and an HFD mimics the fast food diet. CUMS and HFD are two of the main factors that induce depression as well as its comorbidities. However, there are no studies which focus on the treatment of depression based on the TLR4-mediated inflammatory damages in HFD/CUMS-induced depression-like animal models. Puerarin (7,4′-dihydroxyisoflavone-8β-glucopyranoside, Figure 1A) is the major secondary metabolite obtained from the roots of *Pueraria lobata* (Willd.) Ohwi. Traditionally, it is used for the treatment of splenasthenic diarrhea. Recent studies demonstrated that puerarin exerts a wide-spectrum pharmacological effect, such as anti-inflammation, antioxidation, calcium antagonization, reducing blood viscosity, and improving learning and memory in coronary heart disease, diabetes, and neurodegenerative diseases (Zhou et al., 2014; Qiu et al., 2017; Cai et al., 2018; Liu et al., 2019). The signal pathways regulated by puerarin involve TLR4/p38/MAPK, TLR4/NF-κB, PI3K/AKT, cyclic adenosine monophosphate (cAMP), transforming growth factor (TGF)-β1, and GSK-3β/Nrf2 (Ji et al., 2016; Hou et al., 2018; Jeon et al., 2020). Puerarin has been described to treat depression-like animals induced by chronic stress and ovariectomy (Zhao et al., 2017; Tantipongpiradet et al., 2019); however, there is no report on the study of puerarin regulating HFD/CUMS-induced depression. Therefore, the current study aims to evaluate the protective effect of puerarin on HFD/CUMS-induced depressive rats and clarify the molecular mechanisms based on the TLR4-mediated inflammatory responses.

## Materials and Methods

### Materials

Puerarin (>98%) was purchased from Jiangsu Yongjian Pharmaceutical Technology Co., Ltd. (Jiangsu, China); fluoxetine hydrochloride (Prozac) was purchased from Eli Lilly and Company; simvastatin was obtained from Dalian Meilun Biotech Co., Ltd. (Dalian, China); rat ELISA kits of IL-6 and TNF-α were purchased from PeproTech (Rocky Hill, United States); the IL-10 rat ELISA kit was purchased from eBioscience (San Diego, United States); the BCA Protein Assay Kit was purchased from Pierce (Rochford,United States); Goat Anti-Mouse IgG H&L (Alexa Fluor® 647) antibodies were purchased from Abcam (Cambridge, MA); anti-TLR4, anti–claudin-1, anti-occludin, anti–β-actin, and HRP-conjugated Goat Anti-Rabbit IgG antibodies were purchased from Servicebio (Wuhan, China); the UNIQ-10 column TRIzol total RNA extraction kit was obtained from Sangon Biological Engineering Technology & Services Co., Ltd. (Shanghai, China). The FastStart Universal SYBR Green Master (ROX) kit was purchased from Roche (Mannheim, Germany); the calcium-dependent cytosolic phospholipases A2 (cPLA2) assay kit and prostaglandin E_2_ (PGE_2_) Express EIA Monoclonal Kit were purchased from Cayman Chemical (Minneapolis, United States). The COX-2 inhibitor screening kit was obtained from Beyotime Biotechnology (Shanghai, China).

Male Sprague–Dawley (SD) rats (160–180 g) and a high-fat diet containing 4.6 kcal/g (49% fat, 20% protein, and 31% carbohydrate) were purchased from Qingdao Daren Fucheng Animal Husbandry Co., Ltd. [SCXK: (Lu) 2019 0003, Qingdao, China]. The rats were housed in a ventilated, temperature-controlled (22–24°C), and standardized sterile animal room at Jining Medical University. All of the rats were adapted for 1 week with free access to food and water. All animal experiments in the present study were performed according to the National Institutes of Health guidelines for animal care and approved by the Ethics Committee of Jining Medical University (Approval No. 2019-YX-04).

### HFD/CUMS Procedure and Drug Administration

The rats were randomly divided into 10 groups with 10 rats per group, including 1) the normal control group, 2) HFD group, 3) CUMS group, 4) HFD/CUMS group, 5) HFD/CUMS + fluoxetine (10 mg/kg) group, 6) HFD/CUMS + simvastatin (10 mg/kg) group, 7) HFD/CUMS + fluoxetine + simvastatin group, (8–10) HFD/CUMS + puerarin (30, 60, and 120 mg/kg) groups. As shown in Figure 1B, the rats received a HFD except those which were fed with a normal diet in the control and CUMS groups for 11 weeks. All rats received 1% (w/v) sugar water training since the third day of the seventh week for 4 days: two bottles of 1% sucrose per cage for 24 h; one bottle of water and one bottle of 1% sugar water for another 24 h; and two bottles of water for another two days. Since the eighth week, the rats in the CUMS and HFD/CUMS groups were exposed to one random stressor between 9:00 and 11:00 a.m. per day for 4 weeks. The stressors included noises for 3 h (60 dB), tail clamp for 2 min, swimming in cold water (10°C) for 5 min, inversion of light/dark cycle for 24 h, food deprivation for 24 h, water deprivation for 24 h, and level shaking for 5 min (1 time/s). Since the 11th week, the rats in various groups were given sterile 0.9% saline, fluoxetine, simvastatin, fluoxetine + simvastatin, puerarin (30, 60, or 120 mg/kg), respectively, once per day for 7 days. Fluoxetine, simvastatin, and puerarin were dissolved in sterile 0.9% saline solution and stored at 4°C for up to 24 h.

### Open-Field Test, Body Weight Test, and Sucrose Consumption Test

The open-field test was used to explore the behavior of rats. The open-field apparatus was a 100 × 100 × 40 cm black box equipped with a video tracking system (Ethovision, Netherlands). Each rat was placed into the center of the box and was observed for 6 min. The rearing numbers, time resting, time activity, and total distance traveled during the last 4 min were recorded and statistically analyzed.

The body weight of all rats was recorded at days 0, 7, 14, 21, 28, 35, 42, 49, 56, 63, 70, and 77. The 1% sucrose preference test was carried out at days 50, 57, 64, 71, and 78 to evaluate the anhedonic-like state of rats (Pothion et al., 2004). Before the test, all of the rats were deprived of food and water for 24 h and then fed with one bottle of water and one bottle of 1% sucrose solution for 1 h (8:00–9:00 a.m.). Water and sucrose solution consumption was obtained by weighing the bottles before and after the test. The sucrose preference rate was calculated according to the following formula:

$$\mathrm{Sucrose consumption}\left(\%\right)=\frac{\mathrm{Sucrose intake}}{\mathrm{Sucrose water intake + Water intake}}\mathrm{\times 100}.$$

### Detection of Inflammatory Cytokines

After the behavior test, blood of anesthetized rats was collected into heparinized anticoagulant tubes and centrifuged at 3,000 rpm for 10 min. Plasma was then obtained and stored at −20°C until use. Simultaneously, the hippocampal tissues of rats were quickly harvested, frozen in lipid nitrogen immediately, and stored at −80°C. The levels of IL-6, TNF-α, and IL-10 in plasma and hippocampal tissues were detected by commercial ELISA kits, according to the manufacturer’s instructions. The results of hippocampal tissues were corrected as picogram per milligram protein.

### Histological Analysis of Small Intestines

After anesthetization, the rats were killed, and 2 cm of small intestines was dissected and fixed in 10% formalin solution for hematoxylin and eosin (H&E) staining. The histological and pathological changes of each slice were observed by using an optical microscope at a magnification of 100 ✕.

### Western Blotting Analyses

The small intestines and hippocampal tissues (∼50 mg) were quickly harvested, frozen in liquid nitrogen immediately, and stored at −80°C. The protein expression of TLR4, claudin-1, and occludin was determined by Western blotting. Small intestines and hippocampal tissues were homogenized using lysis buffer at a ratio of 1:5 (mg/μl) and quantified by the BCA protein assay kit (Thermo, United States). Samples containing an equal amount of the protein (40 μg) were subjected to 10% SDS-PAGE electrophoresis and blotted on the PVDF membrane. After blocking with 5% bovine serum albumin (BSA) in phosphate-buffered saline-Tween 20 (PBST) for 2 h at room temperature, the PVDF membrane was incubated with respective primary antibodies at 4°C overnight. The dilution of anti-TLR4, anti–claudin-1, anti-occludin, and anti–β-actin antibody was 1:1000, 1:500, 1:1000, and 1:1500, respectively. After washing with PBST, the membrane was incubated with horseradish peroxidase–conjugated secondary antibody (1:3000) for 2 h at room temperature. Then, the membranes were washed five times using PBST and incubated with the ECL substrate. Finally, the membrane was scanned into a computer, and densitometry was quantified using ImageJ software.

### Real-time RT-PCR Analysis for mRNA Expression

Total RNA of hippocampal tissues was extracted using the Sangon UNIQ-10 column TRIzol total RNA extraction kit and reverse-transcribed using the ImProm-II Reverse Transcription System cDNA synthesis kit, according to the manufacturer’s instructions. Primer sequences used for TLR4, occludin-1, claudin, and β-actin are shown in Table 1. The real-time RT-PCR reactions were performed using the SYBR Green system on an ABI 7500 PCR System (Thermo Fisher, MA, United States). MRNA expression was normalized to β-actin mRNA levels. Relative expression was determined relative to the normal control using the 2^−∆∆C_T_^ method (Livak and Schmittgen, 2001).

**TABLE 1:** Real-time RT-PCR oligonucleotide primers.

Gene	Primer	Sequence (5′-3′)	PCR product (bp)
β-actin	Forward	TGT​TAC​CAA​CTG​GGA​CGA​CA	165
(NM_007393.3)	Reverse	GGG​GTG​TTG​AAG​GTC​TCA​AA	—
TLR4	Forward	GAG​CCG​GAA​AGT​TAT​TGT​GG	150
(NM_019178.2)	Reverse	AGC​AAG​GAC​TTC​TCC​ACT​TTC​T	—
Occludin	Forward	GAG​GGT​ACA​CAG​ACC​CCA​GA	161
(NM_031329.3)	Reverse	CAG​GAT​TGC​GCT​GAC​TAT​GA	—
Claudin-1	Forward	CCT​CCA​ATG​CCG​TTC​TGT​AT	118
(NM_031699.3)	Reverse	AGG​GCC​TTT​GCT​ACA​GAT​GA	—

### TLR4 Immunofluorescence Staining

After dissection, the rat brain tissues were immediately fixed in 10% (v/v) neutral formalin solution. Then, the tissues were embedded in paraffin and cut to 5 μm thick. The slides were deparaffinized and rehydrated before immunofluorescence staining using the anti-TLR4 antibody overnight at 4°C. After washing with phosphate-buffered saline (PBS, ph = 7.4), TLR4-positive cell detection was enhanced by using a Goat Anti-Mouse IgG H&L (Alexa Fluor® 647) antibody (Abcam, Cambridge, MA) in the dark. Moreover, the nuclei were stained with 4′,6′-diamidino-2-phenylindole (DAPI, 2 μg/ml). The stained slides were observed on a CRI fluorescence imaging system (Maestro2, CRI, United States) and analyzed using ImageJ software.

### Non-Targeted Lipidomic Analysis and Data Processing

Rat hippocampal tissues (∼80 mg) were transferred into 2-ml centrifuge tubes. A total of 600 μl chloroform methanol mixed solution (2:1, precooled at −20°C) was added and vortexed for 30 s. After transferring the samples to ice for 40 min, 190 μl H_2_O was added and vortexed for 30 s. After transferring the samples on ice for 10 min, the supernatant was centrifuged at 12,000 rpm for 5 min at room temperature. Then, ∼300 μl of the lower layer was transferred into a new contribute tube. This cycle was repeated once, and the samples were concentrated and dried in vacuum. Finally, the samples were dissolved with 200 μl isopropanol, and the supernatant was filtered through a 0.22-μm membrane to obtain the prepared samples for LC-MS.

Separation and chromatography of the samples on an Agilent 1290 infinity series UHPLC (Agilent Corporation, CA, United States) coupled to a Triple TOF® 6600 mass spectrometer system (AB SCIEX, United States)were performed. In brief, chromatographic separation was performed on a Phenomenex Kinetex C18 column (100 × 2.1 mm, 1.7 μm) maintained at 55°C. The temperature of the autosampler was 8°C. Gradient elution of the analytes was carried out with acetonitrile: water = 40:60 (10 mM ammonium formate) (A) and isopropanol: acetonitrile = 90:10 (10 mM ammonium formate) (B) at a flow rate of 0.30 ml/min. The injection volume was 2 μl in the positive mode and 6 μl in the negative mode. An increasing linear gradient of solvent B (v/v) was used as follows: 0–1.5 min, 40% B; 1.5–10.5 min, 40–85% B; 10.5–14 min, 85% B; 14–14.1 min, 85–100% B; 14.1–15 min, 100% B; 15–15.2 min, 100%–40% B; and 15.2–18 min, 40% B.

The mass spectrometry parameters were used as follows: ion source gas 1 (GS1), 60 psi; ion source gas 2 (GS2), 60 psi; curtain gas (CUR), 30 psi; temperature, 600°C; and ion spray voltage floating (ISVF), 5,000 V or −4,500 V in positive or negative modes; declustering potential (DP) 100 V. The data-dependent acquisition (DDA) method was used for MS/MS acquisition. Each acquisition cycle consists of one rapid TOF MS survey scan (200 ms) followed by the consecutive acquisition of 11 product ion scans (50 ms each). For the TOF MS survey scan, the mass range is from 200 to 2,000 Da, and collision energy (CE) is set as 10 V. For the product ion scan, the mass ranges are from 100 to 2,000 Da, and collision energy (CE) is set as 45 ± 25 V. Dynamic background subtraction was applied. Dynamic exclusion was implemented to remove some unnecessary information in the MS/MS spectra.

LipidView software (v4.2) was used to annotate the obtained raw data and then peak identification, filtration, and alignment were performed. The data were normalized by MetaboAnalyst 5.0 (https://www.metaboanalyst.ca/). Principal component analysis (PCA) and partial least square analysis (PLS-DA) were carried out. Differentially altered lipid metabolites were screened based on variable importance in projection (VIP) values. Metabolites with VIP >1 and p (corr) < 0.05 were further evaluated with an independent sample t-test. Moreover, the false discovery rate (FDR) was also used to evaluate the significance of differences in each metabolite. An FDR less than 0.05 was considered as significant.

### Molecular Docking Simulations

The binding ability and binding mode of puerarin to cPLA2 and COX-2 were investigated through molecular docking studies. The crystallography structure of cPLA2 [PDB ID: 1DB5 (Schevitz et al., 1995)] and COX-2 [PDB ID: 3QH0 (Liang, 2011)] was retrieved from the PDB database and preprocessed in BIOVIA Discovery Studio 2017. Puerarin was then docked to cPLA2 and COX-2 with the CDOCKER module in Discovery Studio 2017. The binding site sphere radius was set to 10 Å for COX-2 and 9 Å for cPLA2. Twenty docked poses were generated for each docking simulation. The top-ranked poses were selected for further analysis. 3D figures were generated by Discovery Studio 2017. 2D diagrams were generated by LigPlot v.1.0 (Wallace et al., 1995).

### Enzyme Activity Detection of cPLA2 and COX-2

For the enzyme activity detection of cPLA2, small intestine and hippocampal tissues were perfused with PBS containing 0.16 mg/ml heparin to remove red blood cells and clots. Then, the tissues (50 mg) were homogenized in 1 ml pre-cold buffer (50 mM HEPES, pH 7.4, containing 1 mM EDTA). After being centrifuged, the supernatant was analyzed according to the instructions of the commercial kit (Cayman Chemical, Ann Arbor, MI, United States). The samples were diluted at 1:5 and 1:10, respectively, for hippocampal tissues and small intestines. The absorbance was recorded at 414 nm using a plate reader. To detect the enzyme activity of COX-2, the COX-2 inhibitor screening kit (Beyotime Biotechnology, China) was used. The hippocampal tissues and small intestines were homogenized using lysis buffer at a ratio of 1:5 (mg/μl) and quantified by the BCA protein assay kit (Thermo, United States). The enzyme activity of COX-2 of the samples was analyzed following the instructions of the commercial kit and corrected as Relative Fluorescence Unit (RFU) per gram protein.

### Assay of PGE_2_ Production

Production of PGE_2_ in the hippocampus and small intestine tissues was detected by enzyme-linked immunosorbent assay (ELISA). The hippocampal tissues and small intestines were homogenized using lysis buffer at a ratio of 1:8 (mg/μl) and quantified by the BCA protein assay kit (Thermo, United States). The concentration of PGE_2_ was detected according to the instruction of the commercial PGE_2_ EIA monoclonal kit (Minneapolis, United States). The results were corrected as picogram per milligram protein.

### Statistical Analysis

All data were represented as mean ± S.E.M. N indicated the number of animal samples in each group. Statistical analyses were performed with Origin Pro 2021 software by Student’s t-test (between two groups) or one-way analysis of variance (ANOVA, among multiple groups). The *p*-value less than 0.05 was considered statistical significant.

## Results

### Puerarin Improved Sucrose Preference and Depression-Like Behavior in HFD/CUMS-Induced Rats

**FIGURE 2:**
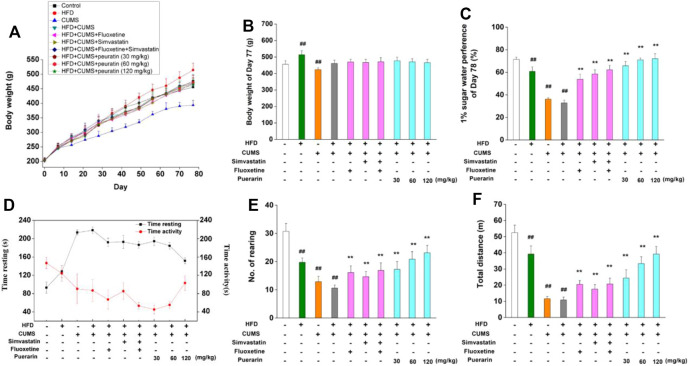
Effect of puerarin on body weight, sucrose preference, and depression-like behavior. **(A)** Body weight of all rats was recorded at days 0, 7, 14, 21, 28, 35, 42, 49, 56, 63, 70, and 77. **(B)** Body weight at the 77^th^ day was statistically analyzed. **(C)** Water and sucrose solution consumption at the 78^th^ day were carried out, and the sucrose preference rate was calculated. The time resting or activity **(D)**, rearing numbers **(E)**, and total distance **(F)** traveled during the last 4 min were recorded and statistically analyzed. Values are expressed as mean ± S.E.M (*n* = 8). ^##^ *p* < 0.01 vs. the normal control group; ^**^ *p* < 0.01 vs. the HFD/CUMS group.

In order to investigate the protective effect of puerarin on depression, an HFD/CUMS-induced depression-like rat model was used. As shown in Figures 2A,B, in comparison with the normal control group, the body weight of rats in the HFD group significantly increased from the 56^th^ day, while significant weight loss was observed in the CUMS-alone group at the 77^th^ day (*p* < 0.01). However, no significant difference was found among other groups because an HFD and CUMS have opposite effects on weight gain. Anhedonia, the main symptom of depressive disorder, was detected by the 1% sucrose preference test. Compared with the normal control group, rats in the HFD, CUMS, and HFD/CUMS groups showed significantly less sucrose consumption (Figure 2C, *p* < 0.01). The drug treatment groups, including simvastatin, fluoxetine, simvastatin + fluoxetine, and puerarin (30, 60, and 120 mg/kg), showed significant higher consumption of sucrose solution than the HFD/CUMS group (*p* < 0.01). In the open-field test (Figures 2D–F), HFD, CUMS, and HFD/CUMS stimulation prolonged time resting, while decreasing time activity and number. of rearing and total distance (*p* < 0.01), showing behaviors indicative of depression. In comparison with the HFD/CUMS group, simvastatin, fluoxetine, simvastatin + fluoxetine, and puerarin (30, 60, and 120 mg/kg) treatments significantly alleviated the depression-like behaviors (*p* < 0.01).

### Puerarin Antagonized the Abnormal Protein Expression of IL-6, TNF-α, and IL-10 in Hippocampal Tissues and Plasma

**FIGURE 3:**
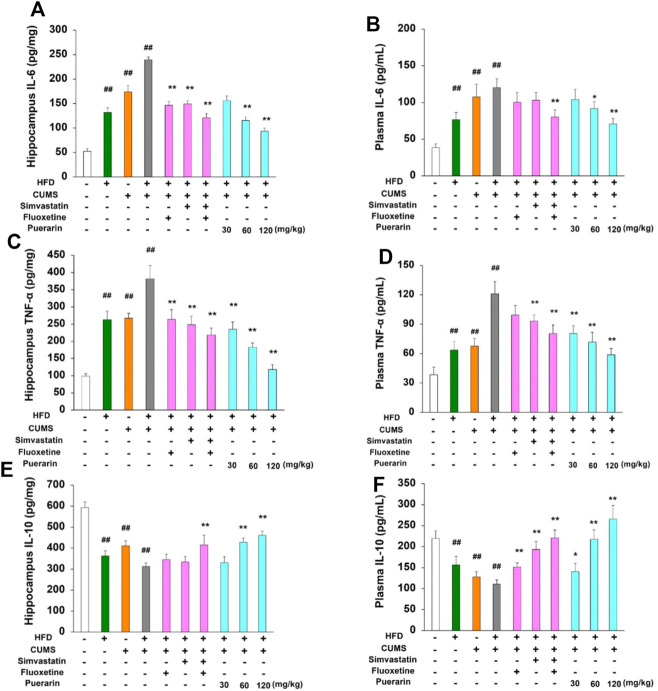
Effect of puerarin on protein expression of IL-6, TNF-α, and IL-10 in hippocampal tissues and plasma. After drug treatment for consecutive 7 days (one time per day), plasma and hippocampal tissues were obtained. The protein levels of IL-6 **(A, B)**, TNF-α **(C, D)**, and IL-10 **(E, F)** in hippocampal tissues and plasma were detected by using commercial ELISA kits, respectively. The results of hippocampal tissues were corrected as picogram per milligram protein. Values are expressed as mean ± S.E.M (*n* = 8). ^##^ *p* < 0.01 vs. the normal control group; ^*^ *p* < 0.05 and ^**^ *p* < 0.01 vs. the HFD/CUMS group.

To further investigate the protective role of puerarin on HFD/CUMS-induced inflammation in the peripheral and CNS, the protein levels of IL-6, TNF-α, and IL-10 in plasma and hippocampal tissues were detected. The results in Figure 3 demonstrated that chronic stimulation of HFD, CUMS, and HFD/CUMS significantly induced inflammatory damage by enhancing the protein level of IL-6 and TNF-α and reducing the expression of IL-10 in the hippocampus and plasma (*p* < 0.01). However, in comparison with the HFD/CUMS group, simvastatin + fluoxetine or puerarin treatment antagonized the abnormal protein expression of IL-6, TNF-α, and IL-10 in hippocampal tissues and plasma (*p* < 0.01 or *p* < 0.05). Moreover, a dose-dependent manner was observed in puerarin-treated (30, 60, and 120 mg/kg) groups.

### Puerarin Repaired Pathological Damage of the Small Intestine of Depression-Like Rats

**FIGURE 4:**
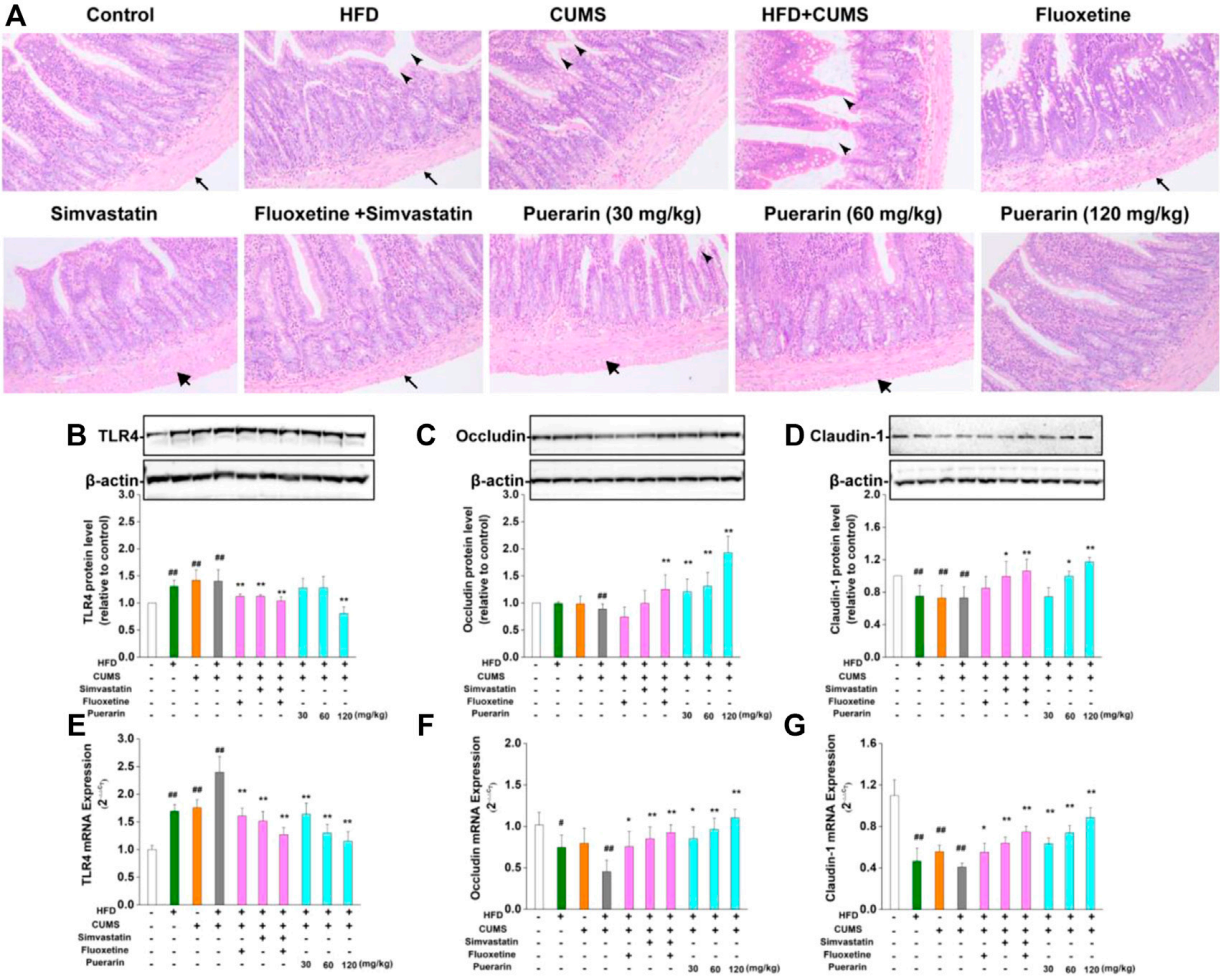
Effect of puerarin on HFD/CUMS-induced small intestine mucosa. **(A)** Pathological damage of the small intestine was detected by H&E staining.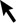 indicates the thickness from the serosa to muscularis;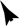 shows that the villi were swollen and broken, and the arrangement of the lamina propria cells was completely disordered;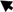 indicates that the serosal layer was loosely arranged or fell off from the muscle layer. The magnification was 100 ×. The protein expression of TLR4 **(B)**, occludin **(C)**, and claudin-1 **(D)** was analyzed by Western blotting. β-actin was used as the internal reference. The densitometry was quantified using ImageJ software. The mRNA levels of TLR4 **(E)**, occludin **(F)**, and claudin-1 **(G)** were detected by real-time RT-PCR. β-actin was used as the housekeeping gene. Values are expressed as mean ± S.E.M (*n* = 3 for Western blotting and *n* = 4 for real-time RT-PCR). ^#^ *p* < 0.05 and ^##^ *p* < 0.01 vs. the normal control group; ^*^ *p* < 0.05 and ^**^ *p* < 0.01 vs. the HFD/CUMS group.

As shown in Figure 4A, in the normal control group, the small intestinal mucosal villi were arranged regularly, and no obvious damage was observed. The small intestinal mucosa of the rats in the HFD, CUMS, and HFD/CUMS groups were severely damaged, including the following characteristics: 1) the thickness from the serosa to muscularis was less; 2) the villi were broad and short and considerably swollen, broken, or necrotic; and 3) the arrangement of the lamina propria cells was completely disordered. Long-term stimulation of CUMS showed that the serosal layer was loosely arranged or fell off from the muscle layer. Among which, the morphological structure of the small intestine mucosa of rats in the HFD/CUMS group was the most severely damaged. Compared with the HFD/CUMS group, the changes of the small intestine mucosa were improved. Moreover, the simvastatin + fluoxetine-treated group and puerarin (120 mg/kg)-treated groups showed that the villi were basically intact, arranged tightly, and the structure of each layer was relatively clear and complete.

The pathological damage of the small intestine may be related to the increase in intestinal permeability mediated by abnormal expression of TLR4. Subsequently, the mRNA and protein expressions of TLR4, occludin, and claudin-1 were determined by real-time RT-PCR and Western blotting, respectively. As displayed in Figures 4B,E, HFD, CUMS, and HFD/CUMS stimulations significantly increased the mRNA and protein concentrations of TLR4 in the small intestine (*p* < 0.01). However, simvastatin, fluoxetine, simvastatin + fluoxetine, and puerarin (30, 60, or 120 mg/kg) treatments significantly downregulated the abnormal TLR4 levels (*p* < 0.01). Moreover, rats in the HFD, CUMS, and HFD/CUMS groups increased intestinal permeability by reducing the mRNA and protein expression of occludin (Figures 4C,F) and claudin-1 (Figures 4D,G, *p* < 0.01 or *p* < 0.05). After the rats received simvastatin, fluoxetine, simvastatin + fluoxetine, or puerarin (30, 60, and 120 mg/kg), the mRNA expressions of occludin and claudin-1 were considerably upregulated (*p* < 0.01 or *p* < 0.05). Similarly, the protein expression of occludin and claudin-1 was significantly enhanced by simvastatin + fluoxetine or puerarin (30, 60, and 120 mg/kg) (*p* < 0.01 or *p* < 0.05). These results indicated that the intestinal permeability was restored.

### Puerarin Decreased the Number of TLR4-Positive Cells in the Prefrontal Cortex of the HFD/CUMS-Induced Rats

**FIGURE 5:**
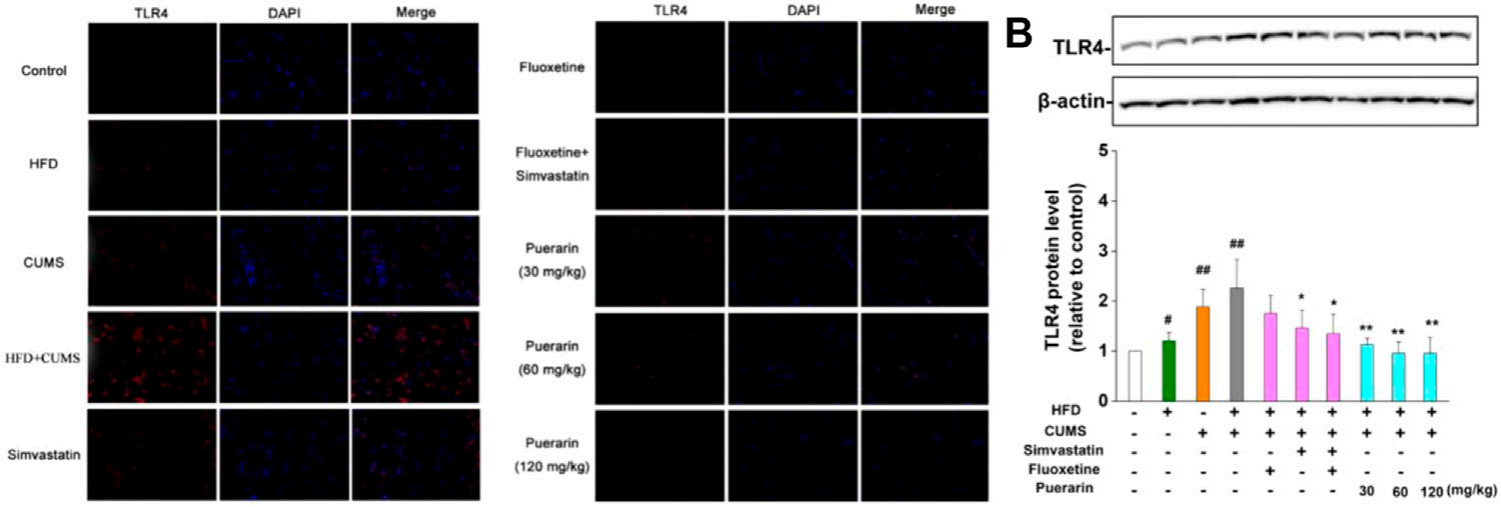
Puerarin decreased the level of CNS TLR4. **(A)** Puerarin decreased the level of TLR4 in the prefrontal cortex of HFD/CUMS-induced depression-like rats (400×magnification). The localization of TLR4 was determined by immunofluorescence. TLR4-positive cells were identified as red, and the nucleus was stained with DAPI in blue. **(B)** Puerarin reduced the expression of TLR4 in the hippocampal tissues of HFD/CUMS-induced depression-like rats. The protein expression of TLR4 was detected by Western blotting. β-actin was used as the internal reference. The densitometry was quantified using ImageJ software. Values are expressed as mean ± S.E.M (*n* = 3). ^#^ *p* < 0.05 and ^##^ *p* < 0.01 vs. the normal control group; ^*^ *p* < 0.05 and ^**^ *p* < 0.01 vs. the HFD/CUMS group.

The TLR4 levels in the prefrontal cortex were identified as red, and the nucleus was stained in blue. As displayed in Table 2 and Figure 5A, the number of TLR4-positive cells in the HFD, CUMS, and HFD/CUMS groups was more than that of the normal control group (*p* < 0.01). Moreover, the intensity of red fluorescence in the HFD/CUMS group was stronger than that of the other groups. In comparison with the HFD/CUMS group, the rats that received fluoxetine, simvastatin, simvastatin + fluoxetine, or puerarin (30, 60, and 120 mg/kg) treatments showed that the number of TLR4-positive cells was reduced to 70.35, 37.78, 30.36, 65.20, 29.64, and 17.02% (*p* < 0.01). Moreover, we detected the expression of hippocampal TLR4. As shown in Figure 5B, HFD, CUMS, and HFD/CUMS stimulations significantly enhanced the expression of TLR4 (*p* < 0.01 or *p* < 0.05). However, rats in the simvastatin, simvastatin + fluoxetine, or puerarin (30, 60, and 120 mg/kg) groups displayed a remarkably decreased TLR4 level (*p* < 0.01 or *p* < 0.05).

**TABLE 2:** Semi-quantification of TLR4-positive cells.

Group	TLR4-positive cells	Groups	TLR4-positive cells
Normal control	1.83 ± 0.31	Simvastatin	8.5 ± 0.99^**^
HFD	8.83 ± 0.6^##^	Simvastatin + fluoxetine	6.83 ± 1.01^**^
CUMS	17.33 ± 1.12^##^	Puerarin (30 mg/kg)	14.67 ± 1.20^**^
HFD/CUMS	22.50 ± 1.31^##^	Puerarin (60 mg/kg)	6.67 ± 0.84^**^
Fluoxetine	15.83 ± 0.87^**^	Puerarin (120 mg/kg)	3.83 ± 0.60^**^

The number of TLR4-positive cells was averaged from six random grids. Values are expressed as mean ± S.E.M (*n* = 6). ^##^ *p* < 0.01 vs. the normal control group; ^*^ *p* < 0.05 and ^**^ *p* < 0.01 vs. the HFD/CUMS group.

### Puerarin Improved Abnormal Lipid Metabolism in Hippocampal Tissues

**FIGURE 6:**
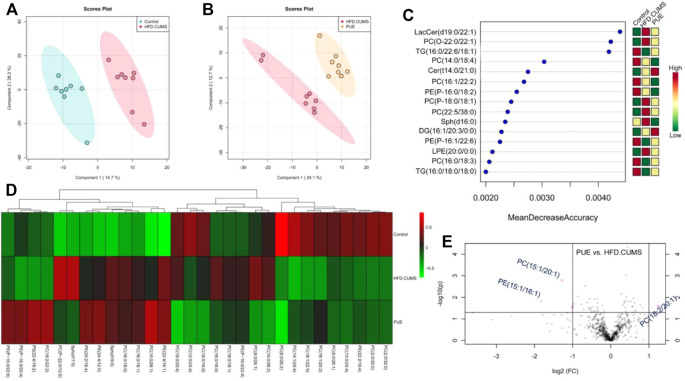
Puerarin-modified lipid metabolism abnormalities in HFD/CUMS-induced rat hippocampal tissues. **(A)** PLS-DA score plot based on the control vs. HFD/CUMS groups. **(B)** PLS-DA score plot based on HFD/CUMS vs. PUE groups. **(C)** Random forest analysis of the top 15 most different metabolites among the control, HFD/CUMS, and PUE groups. “Mean Decrease Accuracy” and “Mean Decrease Gini” were used to measure the importance of a metabolite in discriminating groups in a random forest. The greater the two values, the greater the importance of metabolites in the random forest. **(D)** Heatmap of different phospholipid metabolites. **(E)** Volcano plot based on PUE vs. HFD/CUMS. The identified biomarkers were PC (15:1/20:1), PE (15:1/16:1), and PI (18:2/20:1). LacCer: lactosylceramide, TG: triglyceride, Sph: sphingolipid, PC: phosphatidylcholines, Cer: ceramide, PE: phosphatidylethanolamine, LPE: lyso-PE, DG: diacylglycerols, and PI: phosphatidylinositol. Values are expressed as mean ± S.E.M (*n* = 8).

Lipid metabolites are important signaling molecules in cells. The effect of puerarin on lipid metabolism is shown in Figure 6. PLS-DA score plots showed a complete separation between the control and HFD/CUMS groups and HFD/CUMS and PUE groups, indicating that there were different lipid metabolites among the three groups. The top 15 most different metabolites were LacCer (d19:0/12:1), PC (O-22:2/14:1), TG (16:0/22:6/18:1), PC (14:0/18:4), Cer (14:0/21:0), PC (16:1/22:2), PE (P-16:0/18:2), PC (P-18:0/18:1), PC (22:5/38:0), Sph (d16:0), DG (16:1/20:3/20:0), PE (16:1/22:6), lyso-PE (20:0/0:0), PC (16:0/18:3), and TG (16:0/18:0/18:0). Among the differently altered metabolites, more than 65% were phospholipids. Therefore, based on the phospholipid metabolism abnormalities, the group heatmap is shown in Figure 6D. In comparison with the HFD/CUMS group, the puerarin-modified phospholipid biomarkers were identified as PC (15:1/20:1), PE (15:1/16:1), and PI (18:2/20:1).

### Puerarin Inhibited the Enzyme Activity of cPLA2 and COX-2 and Decreased the Production of PGE_2_

**FIGURE 7:**
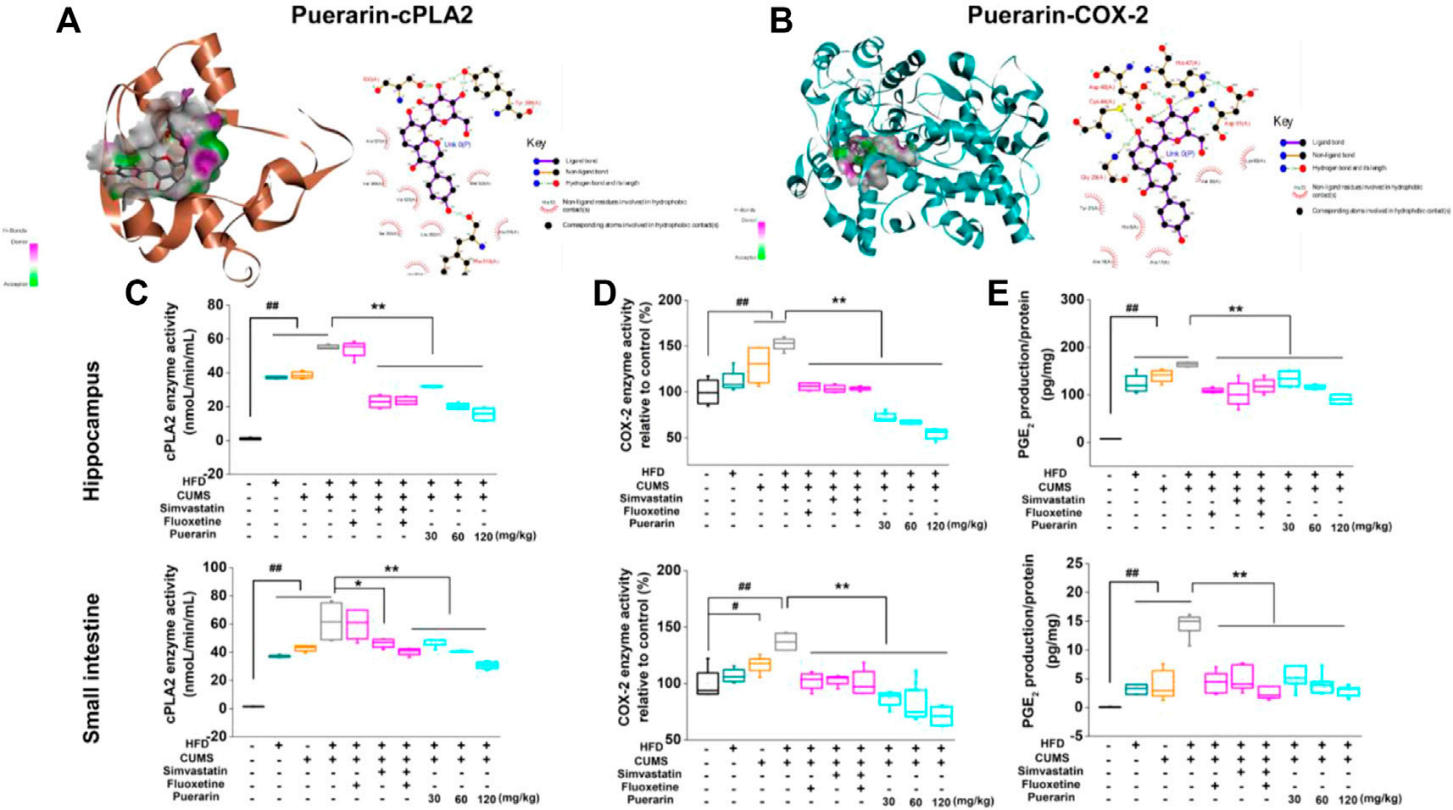
Puerarin inhibited the enzyme activity of cPLA2 and COX-2 and decreased the production of PGE_2_. The 3D and 2D diagrams of the interaction of puerarin-cPLA2 **(A)** and puerarin-COX-2 **(B)** by molecular docking. Puerarin inhibited the enzyme activity of cPLA2 and COX-2 in the hippocampus and small intestine. The enzyme activities of cPLA2 **(C)** and COX-2 **(D)**, as well as the downstream PGE_2_ **(E)** production, were analyzed by using commercial kits. Values are expressed as mean ± S.E.M (*n* = 6). ^#^ *p* < 0.05 and ^##^ *p* < 0.01 vs. the normal control group; ^*^ *p* < 0.05, ^**^ *p* < 0.01 vs. the HFD/CUMS group.

The docking results revealed that puerarin exhibited moderate binding affinity to cPLA2 and weak binding affinity to COX-2 (Figure 7A). The ligand bonded to cPLA2 and COX-2 mainly through hydrogen bonding and hydrophobic interactions. As illustrated in Figure 7B, puerarin formed several hydrogen bonds with Ser530, Tyr385, and Phe518 of COX-2, as well as a number of hydrophobic interactions with Val349, Leu352, and Met522. Puerarin formed hydrogen bonds with Gly29, Cys44, Asp48, and His47 of cPLA2 and hydrophobic interactions with Val30, Tyr21, His6, and Ala17.

To confirm the results of molecular docking, the effect of puerarin on enzyme activities of cPLA2 and COX-2 was evaluated. As shown in Figures 7C–E, HFD, CUMS, and HFD/CUMS stimulation significantly increased the enzyme activities of cPLA2 and COX-2, resulting in enhanced PGE_2_ concentration in hippocampal and small intestinal tissues (*p* < 0.01). In comparison with the HFD/CUMS group, simvastatin, simvastatin + fluoxetine, and puerarin (30, 60, and 120 mg/kg) treatments significantly decreased the enzyme activity of cPLA2 (*p* < 0.05 or *p* < 0.01). Moreover, all drug-treated groups, including fluoxetine, simvastatin, simvastatin + fluoxetine, and puerarin (30, 60, and 120 mg/kg), showed weaker enzyme activities of COX-2 and a lower PGE_2_ level than those in the HFD/CUMS group (*p* < 0.01).

## Discussion

Depression is one of the most common comorbidities of chronic diseases, including cardiovascular, diabetes, and obesity (Bădescu et al., 2016; Lu et al., 2019; Gold et al., 2020). The differential metabolites in urine and plasma between depressive and healthy subjects indicate that the pathogenesis of depressive disorder is related to lipid metabolism, amino acid metabolism, and energy metabolism (Xu et al., 2012; Huang et al., 2020; Wang et al., 2020). Thus, it is necessary to build a complex model to better simulate the multifactorial nature of depression or depression combined with physical diseases. A study based on a single mechanism of depression has certain limitations. A single inducing factor can only simulate one or more pathogeneses of depression (Gururajan et al., 2019). To better explore the pathological mechanism of depression and promote the development of new antidepressant drugs, valid animal models are urgently required. CUMS, as a widely used stimulus for inducing depression-like behavior, reasonably simulates the life state of people who are under long-term mental stress. In terms of nutritional intake, the HFD simulates the unhealthy fast food diet. Thus, the depressant-like model induced by CUMS combined with the HFD may better simulate the complexity of the pathogenesis of depression, including neurotransmitter dysfunction, inflammatory response, abnormal metabolism, and brain–gut axis disorders.

The crosstalk between inflammation and neurocircuits drives the development of depression and has been taken as a potential direction for antidepressant therapies (Miller and Raison, 2016). Cytokines, one of the most investigated immuno-components in depression, directly affect cell functions and communication by exerting pro-inflammatory or anti-inflammatory actions. In addition to central cytokines, peripheral cytokines can, indeed, influence behavior. For example, chronic stress disrupts BBB integrity, promoting peripheral IL-6 to enter into brain parenchyma and resulting in depression-like behaviors (Menard et al., 2017). Cytokines have multiple impacts on neurotransmitter systems. However, it remains unclear how cytokines contribute to the development of depression and what is the underlying mechanism.

In the present study, we evaluated the protective effect of puerarin on HFD/CUMS-induced depression-like rats and explored the molecular mechanisms based on TLR4-mediated inflammatory responses. The results demonstrated that puerarin improved the behaviors in HFD/CUMS-induced depressive rats. IL-6 and TNF-α are two of the most studied pro-inflammatory markers in the blood of major depressive patients (Pedraz-Petrozzi et al., 2020). Following this, we detected the expression of peripheral and central cytokines and found that both HFD and CUMS stimulations heightened the protein concentrations of IL-6 and TNF-α, while blocking the expression of IL-10. However, puerarin treatment significantly alleviated inflammatory damages by restoring HFD/CUMS-induced abnormal levels of IL-6, IL-10, and TNF-α in plasma and hippocampal tissues.

The HFD directly modulates intestinal mucus composition and changes gut microflora by enhancing pro-inflammatory signaling cascades (Rohr et al., 2020). It is unclear if CUMS-induced intestinal hyper-permeability is the pathogenesis or consequence of major depression. However, compelling evidence indicates that CUMS action on the gut may exacerbate neuro-inflammation and neurodegeneration (Dodiya et al., 2020). Therefore, we further detected the histological morphology of small intestine mucosa and the related key molecules, including TLR4, occludin, and claudin-1. HFD and CUMS stimulation showed severe injuries on the villi, such as swelling and breakage. Moreover, the arrangement of lamina propria cells was completely disordered. The difference between HFD and CUMS stimulation is that the former one attenuated the thickness from the serosa to muscularis and the latter one induced the arrangement of the serosal layer loosely or fell off from the muscle layer. However, puerarin (120 mg/kg) treatment significantly restored the intestine mucosa morphology. The villi of the rats which received puerarin (120 mg/kg) were basically intact and arranged tightly, and the structure of each layer was relatively clear and complete. The intestinal hyper-permeability leads to leakiness to endotoxins. TLR4 delivers extracellular antigens into cells and induces inflammatory responses. In addition, TLR4 regulates tight junction proteins, such as occludin and claudin-1 *via* protein kinase C (PKC) hyperactivity (Wardill et al., 2014). Real-time RT-PCR and Western blotting results displayed that puerarin downregulated the mRNA and protein expressions of TLR4, resulting in increased concentrations of occludin and claudin-1. Likely, puerarin-treated groups showed a reduction of TLR4 levels in the rats’ prefrontal cortex and hippocampal tissues. These data indicated that puerarin alleviated peripheral and central inflammatory damages by restoring intestinal permeability *via* TLR4 signaling pathways. Thus, we hypothesized that puerarin exerted antidepressive actions through TLR4-associated mechanisms.

TLR4 regulates COX-2 and PGE_2_, which play an important role in the proliferation and apoptosis in response to intestinal mucosal damage (Fukata et al., 2006). cPLA2 is a key enzyme for membrane phospholipid metabolism. As key enzymes for the metabolism of polyunsaturated fatty acids (PUFAs) and the synthesis of PGE_2_, the genetic variation of cPLA2 and COX-2 genes increases the risk of depression (Su et al., 2010). TLR4 accelerates phospholipid metabolism by activating cPLA2 to produce a large amount of arachidonic acid and free fatty acids, which leads to membrane channel activation and hemodynamic changes (Teply et al., 2016). To further explore the underlying mechanism, non-targeted lipidomics of hippocampal tissues was detected. Among the top 15 most different metabolites, 60 % were phospholipid metabolites. Thus, we mainly focused on the effect of puerarin on phospholipid metabolism. Compared with the HFD/CUMS group, three biomarkers were identified, namely, PC (15:1/20:1), PE (15:1/16:1), and PI (18:2/20:1). cPLA2 and COX-2 are two important enzymes in the process of phospholipid metabolism. Furthermore, molecular docking of puerarin-cPLA2 and puerarin-COX-2 was performed. Although the docking results indicated that puerarin exhibited moderate binding affinity to cPLA2 and weaker binding affinity to COX-2, we verified the results *in vivo* and found that puerarin significantly inhibited the enzyme activities of cPLA2 and COX-2 and decreased the production of PGE_2_ in the small intestine and hippocampal tissues in a dose-dependent manner.

## Conclusion

**FIGURE 8:**
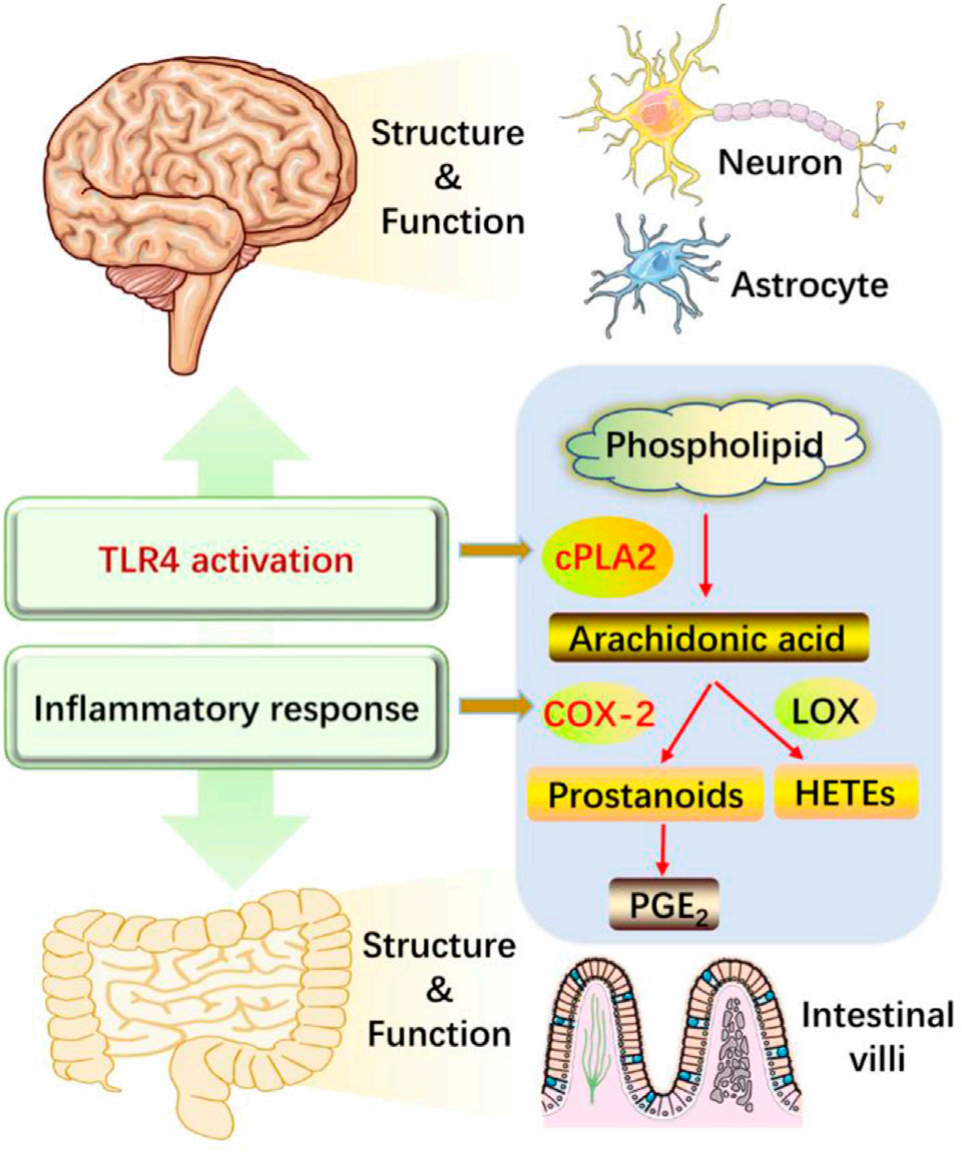
Puerarin protected against HFD/CUMS-induced depression *via* the TLR4/cPLA2/COX-2 pathway. HFD/CUMS stimulation induced depression-like behavior *via* increasing inflammatory damages. Inflammatory response is related to TLR4 activation, inducing the changes of structure and function in the brain and small intestine tissues. Puerarin treatment alleviated the depressive phenotype by suppressing TLR4 activation and cytokine over-production, restoring lipid metabolites, inhibiting enzyme activities of cPLA2 and COX-2, and decreasing downstream PGE_2_ production. HFD: high-fat diet; CUMS: chronic unpredictable mild stress; TLR4: Toll-like receptor 4; LOX: lipoxygenase; cPLA2: calcium-dependent cytosolic phospholipases A2; COX-2: cyclooxygenase-2; PGE_2_: prostaglandin E_2_; HETEs: hydroxyeicosatetraenoic acids.

As illustrated in Figure 8, long-term HFD/CUMS stimulation evokes peripheral and central inflammation responses and TLR4 activation. The abnormal concentrations of inflammatory cytokines change the structure and function of the brain and small intestine, resulting in a depressive phenotype. The interaction between TLR4 and cytokines is complex and is difficult to evaluate if TLR4 activation is the cause or consequence of cytokine outburst. The main concern of the current study is that both TLR4 and cytokines regulate the metabolism of arachidonic acids. Puerarin treatment alleviated HFD/CUMS-induced depression-like behavior by inhibiting TLR4-associated inflammatory responses. Mechanistically, puerarin treatment restored the lipid metabolism abnormalities. For the phospholipid metabolism, puerarin not only changed the phospholipid metabolites but also inhibited the enzyme activities of cPLA2 and COX-2. In conclusion, puerarin treatment reversed HFD/CUMS-induced depression-like behavior by inhibiting TLR4-mediated inflammatory damages and phospholipid metabolism disorders. Subsequently, we will further detect the effects of puerarin on phospholipid metabolism and intestinal microflora to declare the TLR4 signaling pathway–associated functions of the brain–gut axis or other potential mechanisms.

## Data Availability

The original contributions presented in the study are included in the article/Supplementary Material, further inquiries can be directed to the corresponding author.
